# Effects of Attentional Bias Modification on residual symptoms in depression: a randomized controlled trial

**DOI:** 10.1186/s12888-019-2105-8

**Published:** 2019-05-08

**Authors:** Rune Jonassen, Catherine J. Harmer, Eva Hilland, Luigi A. Maglanoc, Brage Kraft, Michael Browning, Tore C. Stiles, Vegard Ø. Haaland, Torkil Berge, Nils Inge Landrø

**Affiliations:** 1 Clinical Neuroscience Research Group, Department of Psychology, University of Oslo, Oslo, Norway; 2 Psychopharmacology and Emotional Research Lab (PERL), Department of Psychiatry, University of Oxford, Oxford, UK; 3 Division of Psychiatry, Diakonhjemmet Hospital, Oslo, Norway; 4 NORMENT, KG Jebsen Centre for Psychosis Research, Division of Mental Health and Addiction, Oslo University Hospital & Institute of Clinical Medicine, University of Oslo, Oslo, Norway; 5 Computational Psychiatry Laboratory, Department of Psychiatry, University of Oxford, Oxford Health NHS Trust, Oxford, UK; 6 Department of Psychology, Norwegian University of Science and Technology (NTNU), Trondheim, Norway; 7 Department of Psychiatry, Sørlandet Hospital, Kristiansand, Norway

**Keywords:** Attentional bias modification, Depression, Clinical trial

## Abstract

**Background:**

Following treatment, many depressed patients have significant residual symptoms. However, large randomised controlled trials (RCT) in this population are lacking. When Attention bias modification training (ABM) leads to more positive emotional biases, associated changes in clinical symptoms have been reported. A broader and more transparent picture of the true advantage of ABM based on larger and more stringent clinical trials have been requested. The current study evaluates the early effect of two weeks ABM training on blinded clinician-rated and self-reported residual symptoms, and whether changes towards more positive attentional biases (AB) would be associated with symptom reduction.

**Method:**

A total of 321 patients with a history of depression were included in a preregistered randomized controlled double-blinded trial. Patients were randomised to an emotional ABM paradigm over fourteen days or a closely matched control condition. Symptoms based on the Hamilton Rating Scale for Depression (HRSD) and Beck Depression Inventory II (BDI-II) were obtained at baseline and after ABM training.

**Results:**

ABM training led to significantly greater decrease in clinician-rated symptoms of depression as compared to the control condition. No differences between ABM and placebo were found for self-reported symptoms. ABM induced a change of AB towards relatively more positive stimuli for participants that also showed greater symptom reduction.

**Conclusion:**

The current study demonstrates that ABM produces early changes in blinded clinician-rated depressive symptoms and that changes in AB is linked to changes in symptoms. ABM may have practical potential in the treatment of residual depression.

**Trial registration:**

ClinicalTrials.gov ID: NCT02658682 (retrospectively registered in January 2016).

**Electronic supplementary material:**

The online version of this article (10.1186/s12888-019-2105-8) contains supplementary material, which is available to authorized users.

## Background

A wide range of treatments are available for depression. However, systematic reviews point to limited efficacy in terms of remission, response rates and long-term effects for both pharmacological [1] and psychological treatments [2]. Forty percent of patients do not, or only partially, respond to treatment, and less than one third are completely recovered after treatment [3]. Residual symptoms following treatment are associated with decreased return to function, reduced quality of life and increased risk of recurrence highlighting the need for further intervention [4].

Depression is associated with an increased focus on negative interpretations of events and negative biases in attention and memory. Previous studies have reported that clinically depressed subjects orient their attention toward negative faces rather than neutral or positive faces [5]. Biases towards negative faces have also been reported in previously depressed, currently euthymic subjects [6], and in never-depressed individuals with a family history of depression [7]. In a seminal experimental study a causal role for negative attention biases in the expression of depressive and anxious symptoms was demonstrated [8]. They found higher levels of anxiety and depression related mood ratings in undergraduates, as a reaction to a stressor, after being trained to preferentially attend to negative versus neutral stimuli. Together these results indicate that negative attentional biases (AB) may constitute causative, vulnerability and maintenance factors rather than simple markers of lowered mood. This suggests that interventions designed to reduce AB may act to reduce symptoms in patients treated for depression.

Computerized Attention Bias Modification (ABM) procedures aim to shift emotional biases towards more positive and less negative stimuli. ABM is low on resource requirements and easy to disseminate and might therefore be widely used by patients with residual symptoms or while waiting for more resource intensive interventions like psychotherapy. While some studies have reported an effect of ABM in depression a number of meta-analyses have suggested a small effect size, although definitive conclusions have been limited by small sample sizes and poor trial methodology employed in many studies [9–11]. In addition, some potential techniques for altering AB have failed to modify AB as part of the treatment programme, which limits their ability to decrease depressive symptoms [12], but see also [13].

The main objective of our study was to test the early efficacy of ABM in a large group of previously depressed patients, but with residual symptoms using a preregistered trial design. We hypothesized that two weeks of ABM training would reduce clinician- and self-reported residual symptoms of depression, as compared to a matched control condition. Given the clear mechanistic role for altered bias in the clinical effects of ABM, we predicted a change towards more positive biases in the ABM group and that this change would be associated with decreased symptoms.

## Methods

### Study design and procedure

Patients who had previously been identified as suffering from depression were randomised to receive two weeks of either ABM- or placebo training. Participants completed two sessions of ABM daily using laptops provided by the research team. A detailed calendar was used to specify the scheduling of the training sessions for each participant. Symptoms of depression and AB were assessed at baseline and immediately after the intervention.

### Participants

The main recruitment base was an outpatient clinic in the Department of Psychiatry, Diakonhjemmet Hospital in Oslo. Participants were also recruited from other clinical sites, by local advertisements, and via social media. Candidates were pre-screened by phone for exclusion criteria before in person formal clinical evaluation and enrollment. Individuals diagnosed with current- or former neurological disorders, psychosis, bipolar spectrum disorders, substance use disorders, attention deficit disorder, and head trauma were excluded.

A total of 377 participants between 18 and 65 years old were recruited for clinical evaluation. Participants had experienced more than one previous episode of depression as defined using the Mini International Neuropsychiatric Interview, version 6.0.0. (M.I.N.I). A total of 56 subjects were excluded following the clinical evaluation. The period of recruitment and follow-up was January 2015 to October 2016 and was first posted in *ClinicalTrials.gov* in January 2016, and was therefore retrospectively registered as registration took place after the first participant was enrolled.

A total of 321 participants with a history of Major Depressive Episodes (MDE) were randomised to receive ABM or placebo (Table 1). After formal diagnostic evaluation, thirty-seven participants also fulfilled the M.I.N.I criteria for a current MDE. The study used an Intention to treat (ITT) approach, meaning that we included everyone who was recruited, including those people who were recruited incorrectly (which is a protocol violation).

### Attention Bias modification procedure

The ABM task was a computerized visual dot-probe procedure adopted from [14]. Paired images of faces (the stimuli), were presented followed by one or two dots (a probe), which appeared in the same screen location where one of the two faces was previously exposed. Participants were required to press one of two buttons as quickly as possible to indicate the number of dots in the probe. The types of stimuli used during the ABM task were pictures of emotional faces of three valences; positive (happy), neutral, or negative (angry and fearful). A single session of the task involved 96 trials with equal numbers of the three stimulus pair types. In addition, there were equal numbers of trials in which the stimuli were randomly presented for 500- or 1000 ms before the probe was displayed. In each trial of the task, stimuli from two valences were displayed, in one of the following pairing types: positive-neutral, positive-negative, and negative-neutral. In the ABM condition, probes were in the same locus as the more positive/less negative stimuli in 87% of the trials, as opposed to 13% with probes in the same locus as more negative/less positive stimuli. Thus, when completing the ABM, participants should learn to deploy their attention toward the relatively more positive stimuli, without being explicitly aware of allocation to intervention, and in this way develop a more positive AB. The neutral ABM control (placebo) condition was identical in every respect, other than the probe location, that had the same locus as the more positive/less negative 50% -, and same locus as more negative/less positive stimuli in 50% of the trials. Participants completed two sessions of ABM at home daily during the course of fourteen days (28 sessions in total) on identical notebook computers that were set up and used exclusively for ABM-training.

### Measurement of attentional Bias

The AB assessment task was identical to a single session of the placebo-training task but used novel face stimuli. Attentional biases (AB) was calculated as the difference in reaction time in milliseconds (RT) between trials in which the probe replaced the relatively more negative face vs. the more positive face ([(SUM (more positive face in upper screen position - locus of probe in lower screen position, more positive face in lower screen position, locus of probe upper screen position) – SUM (more positive face in upper screen position - locus of probe in upper screen position, more positive face in lower screen position- locus of probe in lower screen position)]/2.). Thus a more positive score reflects a greater bias towards the more positive stimuli. The ABM assessment task was conducted in the same lab environment for all participants.

### Randomization and blinding

An independent lab technician (not involved in the day to day collection of data) prepared training computers to deliver either ABM or placebo treatment according to a randomization list in a 1:1 ratio ensuring that allocation was concealed from all researchers involved in screening procedures and all participants. Allocation to intervention is stored in the ABM raw data file that was opened and merged with screening data for statistical analyses first after the data collection period was finished in October 2016. Outcome assessors and participants were therefore blind to allocation during the whole study.

### Study outcome

The primary outcome measures were The Hamilton Rating Scale for Depression (HRSD) [15] and Beck Depression Inventory (BDI-II) [16] administered to assess both clinician - and self-evaluations of symptoms. Secondary outcome was AB measured by the AB assessment task at baseline and after two weeks follow-up. Comorbid anxiety symptomatology was screened by the use of The Beck Anxiety Inventory (BAI-II) [17].

### Statistical analysis

All data were analysed using PASW 25.0 (IBM). The primary outcomes for the ITT sample was analysed by using repeated measures ANOVA with intervention (ABM versus placebo) as a fixed factor. Symptom at baseline and at two weeks follow-up (HRSD or BDI-II) was entered as within subject variable (time). To test the interaction between changes in symptoms and changes in AB, statistically significant symptom changes between ABM and placebo were followed up by repeated measures ANOVA by adding AB (two weeks follow-up minus baseline across valences and stimulus durations) as a second time factor in the model. Thereafter, the individual distribution of symptom change and AB was explored within groups with linear regression models.

A requirement of the national ethical committee was that study participants could elect to have all data stored on them during the study erased (i.e. including randomisation information) which precludes imputation of missing data. Of the 20 participants who did not complete the study following randomisation, 10 elected to have their data erased and thus data was imputed only for the remaining 10 remaining allocated to intervention. Thus, Thus the ITT analyses included 311 participants while participant with full data sets included 301 participants (see Additional file 1: Figure S1). A detailed description of procedures for data reduction, imputation and a post hoc analysis of the potential impact of noncompliance, stimulus valence- and duration, and sensitivity analysis are provided as Additional file 2.

**Table 1:** Demographic and sample characteristics (means (SD) or number). MDE = Major Depressive Episodes according to M.I.N.I. SSRI = participants currently using an antidepressant belonging to the Selective Serotonin Reuptake Inhibitors. ISCED = International Standard Classification of Education. *P*-values from Pearson Chi-Square test are presented for dichotomous variables

	Placebo (*n* = 148)	ABM (*n* = 153)	*p*-value
Age	41.5 (13.6)	40.2 (12.7)	.68
Gender (females)	103	109	.68
Education Level (ISCED)	5.9 (1.2)	6.0 (1.1)	.79
Medication (current SSRI)	43	38	.43
Number of previous MDE	4.1 (4.6)	4.1 (4.9)	.92
Symptoms at baseline:			
BDI-II	13.8 (9.7)	14.9 (10.5)	.23
HRSD	8.3 (5.0)	9.2 (5.9)	.12
BAI-II	9.0 (7.4)	9.6 (9.4)	.54

## Results

### Sample characteristics

#### Primary outcomes

**Fig. 1:**
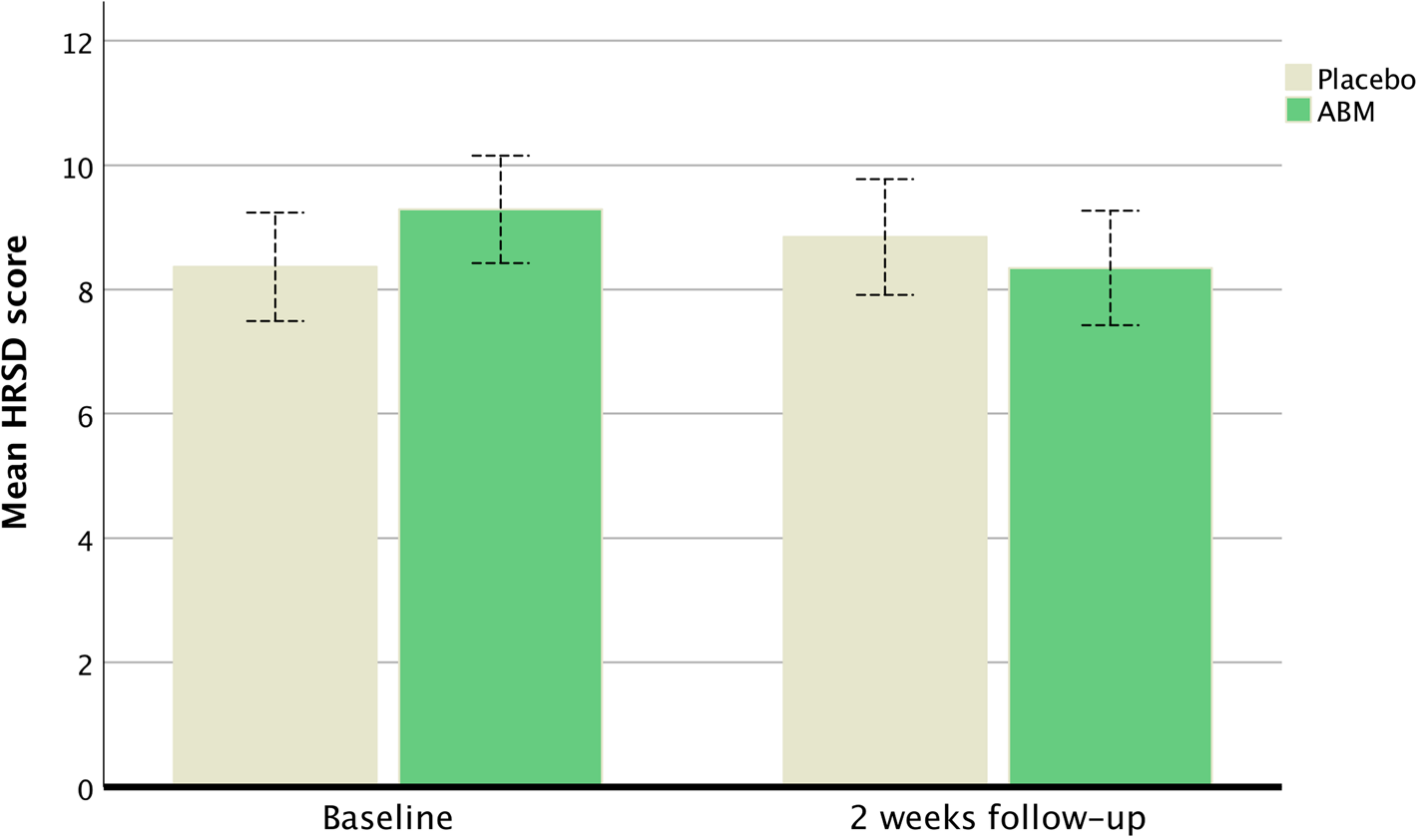
The mean symptom change at baseline and after two weeks of ABM training for HRSD. Whiskers represent the 95% CI

There was a statistically significant difference (time) between ABM and the control condition in blinded clinician-rated symptoms as measured by the HRSD [F (1,309) = 6.78, η^2^ = .02, *p* < .01]. Means and standard deviations at baseline were 9.2 (5.9) for ABM and 8.3 (5.0) for placebo and changed to 8.3 (5.9) for ABM and 8.8 (5.7) for placebo at two weeks follow-up. There was no statistically significant pre-post effect (time) across ABM and Placebo [F (1,309) = .717, *p* = 39]. Together the results show a positive change after ABM combined with symptom worsening in the placebo group at two weeks follow-up. A post hoc repeated ANOVA within groups showed that the observed mean decrease in HRSD scores was statistically significant for the ABM group [F (1,156) = 5.542, η^2^ = .03, *p* = .02], but that the mean increases of HRSD scores was not statistically significant [F (1,153) = 1.667, *p* = .19] (Fig. 1).

Although not statistically significant, there was a mean HRSD difference at baseline A post hoc sensitivity test was therefore conducted by excluding potential outliers in the high end of HRSD scores (HRSD > 17 = cut-off for moderate depression). The post hoc test also showed a statistical significant difference (time) between ABM and the control condition in blinded clinician-rated symptoms as measured by the HRSD. Means and standard deviations at baseline were 7.5 (3.9) for ABM and 7.2 (3.9) for placebo and changed to 7.3 (5.1) and 8.1 (5.4) at two weeks follow-up [F (1,272) = 4.48, η^2^ = .02, *p* = .03] indicating that the observed changes in clinician-rated symptoms was not confounded by random variation at baseline or by patients with the highest symptom scores at baseline.

**Fig. 2:**
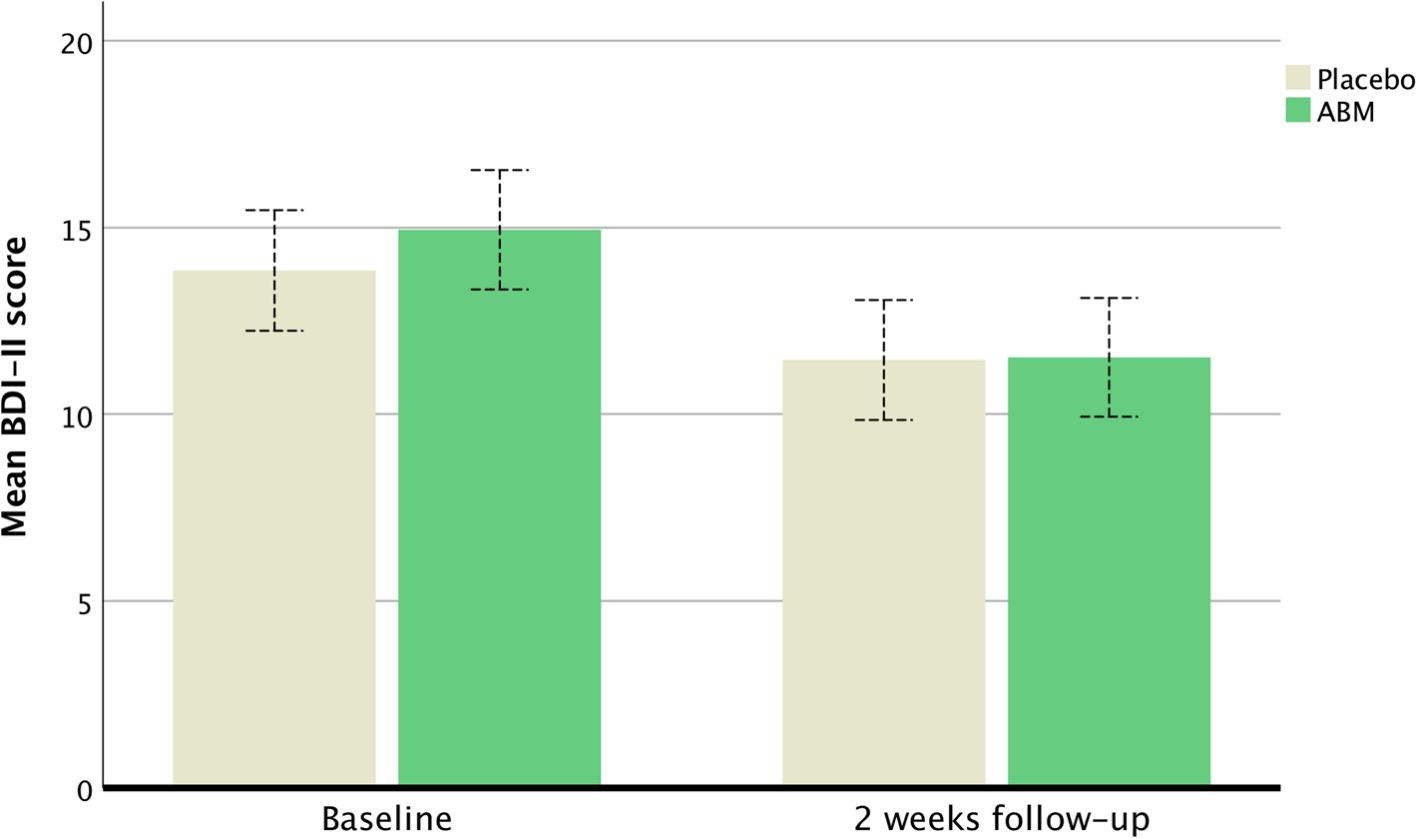
The mean symptom change at baseline and after two weeks of ABM training for BDI-II. Whiskers represent the 95% CI

There was no statistically significant difference in self-reported depression as measured by the BDI-II between the ABM and the control condition [F (1,309) = 2.07, *p* = .15]. Both ABM and placebo showed statistically significant (time) improvement based on the BDI-II [F (1,309) = 67.77, η^2^ = .18, *p* < .001]. Means and standard deviations at baseline were 14.9 (10.5) for ABM and 13.8 (9.7) for placebo and were 11.5 (10.4) and 11.4 (9.8) at two weeks follow-up A post hoc repeated ANOVA within groups showed that the observed mean decrease in BDI-II scores was statistically significant for the ABM group [F (1,156) = 22.945, η^2^ = .13, *p* < .001], and also for the placebo group [F (1,153) = 47.048, η^2^ = .23, p < .001] (Fig. 2).

#### Symptom change after ABM and attentional biases

**Fig. 3:**
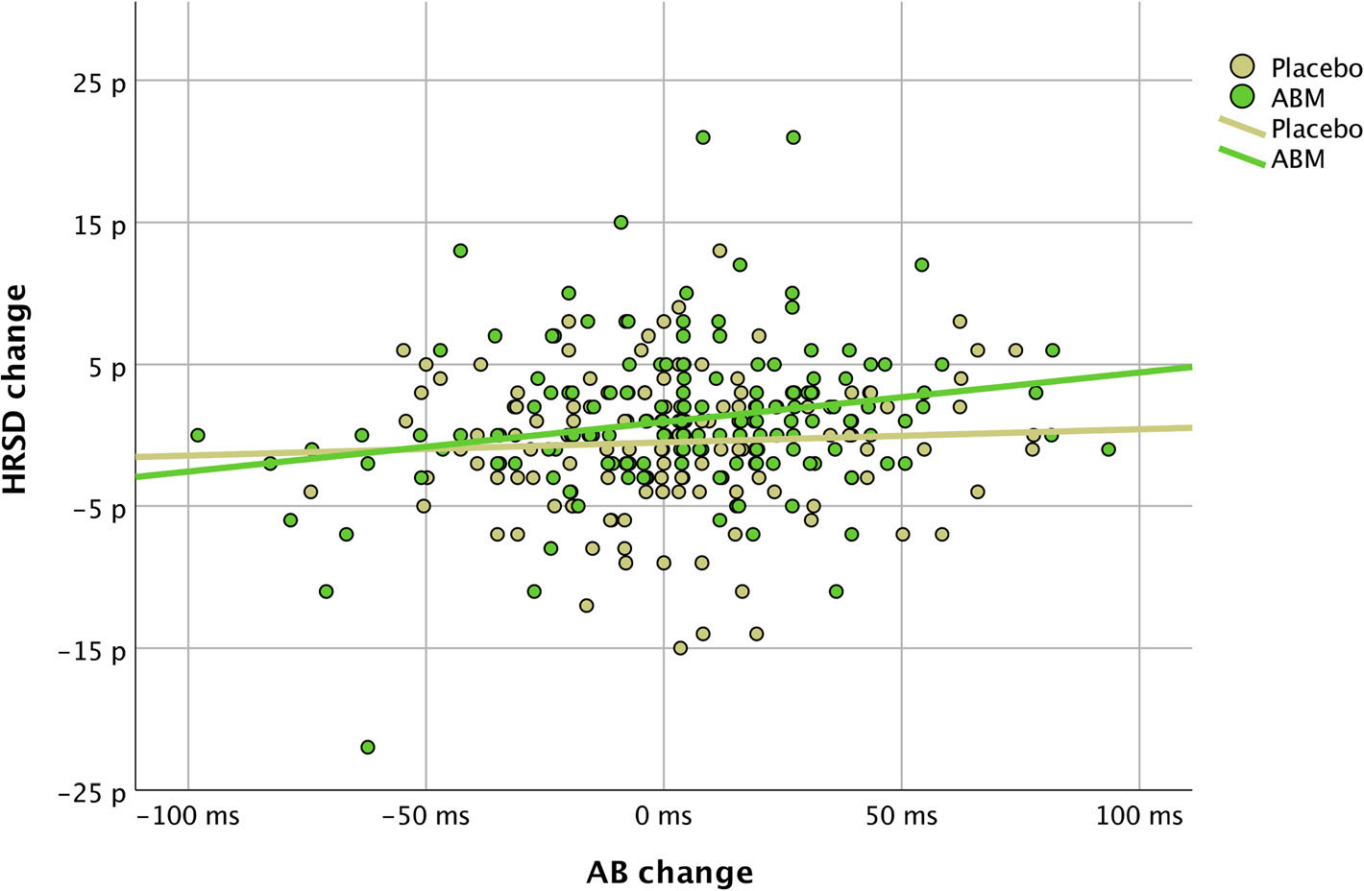
Relationship between changes in HRSD (baseline minus two weeks follow-up) and changes in AB (two weeks follow-up minus baseline). Positive values = changes towards more positive biases (AB change) and symptom improvement (HRSD change)

A repeated measure ANOVA with AB (time) as the single factor did not reveal mean differences in change between ABM and placebo [F (1,309) = .084, *p* = .77]. A combined factor repeated measure ANOVA revealed a statistically significant interaction between changes in AB (time) and changes in HRSD (time) meaning that an association between relatively more positive biases was associated with symptom change in both groups [F (1,309) = 3.793, η^2^ = .01, *p* = .05]. A post hoc linear regression model showed that there was a statistically significant positive association between HRSD improvement and positive changes and AB within the ABM group (Beta = .22, *p* < .00, R^2 = .05) but not within the placebo group (Beta = .06, *p* = .48) (Fig. 3).

#### Symptom specificity

No statistically significant differences (time) were found for self-reported anxiety as measured by the BAI-II [F (1,309) = 1.648, *p* = .20]. As found for self-reported depression (BDI-II) general symptom improvement was found in both ABM and in the placebo group [F (1,309) = 27.783, η^2^ = .08, *p* < .01].

## Discussion

### Main findings

Two weeks of ABM training significantly reduced blinded clinician-rated symptoms as compared to a control condition in a group of patients previously treated for depression and with various degrees of residual symptoms. Relatively more positive change in AB was associated with symptom improvement. ABM versus placebo did not differentiate changes in AB without taking symptom changes into account, meaning that a beneficial effect of ABM despite no intervention dependent change in AB was found. This means either than ABM is working by some other mechanism or that the measurement of AB is not very robust. Corroborating cognitive models of emotional disorders, the degree of symptom improvement increased with degree of relatively more positive bias across groups. AB change may therefore be a useful clinical measure since it is sensitive to individual differences in clinician rated symptoms, and may predict treatment outcome.

A larger intervention independent effect was also found for self-reported depression and self-reported anxiety. Post hoc exclusion of participants with high HRSD scores had no impact on the ABM effect, and further analyses showed that the results are robust against imputations and compliance rates.

In addition to being among the strongest predictors for recurrence in depressive disorder [4] residual symptoms also cause significant functional impairments, manifested in a variety of domains, including work and leisure activities [18]. The latter is important because one aim of depression treatment is to restore the patients’ previous level of functioning. However, residual symptoms in depression have traditionally not been the target of treatment trials. Given the recurrent nature of the illness, we suggest that this should change and that our study provides an example of how this may be done.

It is not clear why ABM versus placebo did not differ as measured by the BDI-II self-report scale, but instead showed a relatively large improvement in both the ABM- and placebo. In another recent study, fifty-two participants in a major depressive episode (MDE) was recruited for ABM training and a similar significant change in BDI-II in both the ABM- and placebo was found [19]. The lack of association between ABM and self-reported symptoms was not expected from the pre-registered hypothesis and interpreting the source of this lack of evidence is speculative. However, self-report may be more influenced by placebo or expectation effects. A general effect of the intervention may also be linked to cognitive training effects present in both training conditions. In contrast to general improvement in self-reported symptoms, increased depression was found within the placebo group for clinician rated depression. Further explorations of this pattern showed that a HRSD change was only statistically significant within the ABM group, indicating that the negative changes within the placebo group did not drive the observed ABM effect. The intervention may target the exact thing that worsens in the placebo group, but may also improve something else and thereby influence different domains that cancel each other out. Hereunder, clinical assessment might drive the participants’ attention towards depression symptoms and both positive- and negative assessment effects could therefore influence the change patterns in ABM and placebo. An assessment only group will help in distinguishing assessment effects- from ABM in future studies.

Symptom assessment based on both syndrome diagnoses, self-rated and blinded clinician-rated symptoms represent strength in the current study and the results clearly underpin the importance of comprehensive clinical evaluations in future research. The current study used a training task that earlier has shown to reduce self-rated symptoms first after 4 weeks [14]. The sample size (statistical power) and sample characteristics, like degree of residual symptoms, may explain why the current study found intervention dependent symptom changes immediate after training.

Participants completed the training in their homes, which is an advantage as it makes it more feasible as a treatment. Furthermore, compliance was high (see Additional file 2) which is important and contrary to the conclusion in a recent review of meta-analyses [11]. These authors conclude that ABM paradigms are most effective when delivered in the laboratory rather than at home. Specifying the scheduling of the training sessions individually might have increased motivation to do and focus on the task.

### Implications

The results suggest that ABM does indeed exert an effect on blinded clinician-rated symptoms. The small effect sizes could be considered clinically non significant in treatment trials. However, it is not clear how to interpret clinical relevance of small HRSD score changes in this group. The HRSD and BDI-II is only moderately correlated and may partly reflect different depression constructs [20]. It is unclear whether the effect of ABM would increase and generalize across self-rated and clinician-rated symptoms if treatment continued for longer. Although residual symptoms are predictive of risk of relapse, it will be important to test in longer-term prevention studies whether the observed beneficial effect of ABM could translate into clinical relevance like reduction of relapse.

There is a pressing need to improve treatment and thus clinical trials should focus not only on efficacy, but also on identification of the underlying mechanisms through which treatments operate [21]. We observed a number of people with large changes in AB that go along with symptoms improvement, but also with no change in HRSD or even worsening of HRSD scores. Identification of this individual variability may be useful in evaluations of treatment efficacy and in personalized treatment. The current study reports the pre-registered primary outcome, but stratification based on degree of AB, changes in dispersion and/or distribution of AB, or paradigms sensitive to the temporal expression of AB may help advance basic knowledge on the conditional benefit of ABM [22].

### Limitations

The study has several limitations that should be mentioned. Sparse research on this population impede calculations of sample sizes from prior studies but would further increase the stringency of the study design in accordance with CONSORT guidelines [23]. Inclusion of 37 patients that also fulfilled the formal criteria for current depression represents a deviation from the pre-registered protocol. However, sensitivity analyses that excluded participants at the higher end of symptom scores did not explain the results and increases the generalizability of the reported findings. Residual depression was widely defined as the study did not assess symptom change during former treatment and classify all symptoms as residual independent of treatment response or time since the last episode. The stimuli used for ABM training- and AB assessment was adopted from earlier studies and this combination of stimuli was not cross-balanced or validated based on ratings of stimulus valence- and arousal.

## Conclusion

Previous studies have reported a mixture of positive and negative findings [14, 24–26] leading to a degree of scepticism about the impact of ABM on symptoms of depression [9]. Our study is by far the largest randomized controlled clinical trial of ABM in a depressed population and gives a broader and more transparent picture of the true advantage of the intervention in this population. The results verify the proof of principle by showing that changes in AB is linked to changes in symptoms and suggest that ABM may have potential in the treatment of residual depression.

## Additional files

Additional file 1: **Figure S1.** Flow diagram for enrolment, allocation to active placebo or ABM, follow-up after two weeks, and analyses in accordance with CONSORT (28). Not meeting inclusion criteria = no MDE according to M.I.N.I. Other reasons (*n* = 42) = current or former mania and/or hypomania according to M.I.N.I. Ten participants (5 in each group) elected to have their data erased. (DOC 72 kb)

Additional file 2: Supplemental information. (DOCX 22 kb)

## Abbreviations

AB: Attentional Bias
ABM: Attentional Bias Modification
BAI-II: The Beck Anxiety Inventory
BDI-II: Beck Depression Inventory II
HRSD: Hamilton Rating Scale for Depression
ITT: Intention To Treat
M.I.N.I 6.0.0: Mini International Neuropsychiatric Interview
MDD: Major Depressive Disorder
MDE: Major Depressive Episodes
RCT: Randomised Controlled Trials
RT: Reaction Time in milliseconds
