# Supplementary material for: Effects of Attentional Bias Modification on residual symptoms in depression: a randomized controlled trial
Source: BMC Psychiatry. 2019 May 8;19:141. doi: 10.1186/s12888-019-2105-8 (PMC6505271; doi:10.1186/s12888-019-2105-8)
Supplement: Supplementary file 1 — Figure S1. Flow diagram for enrolment, allocation to active placebo or ABM, follow-up after two weeks, and analyses in accordance with CONSORT (28). Not meeting inclusion criteria = no MDE according to M.I.N.I. Other reasons (n = 42) = current or former mania and/or hypomania according to M.I.N.I. Ten participants (5 in each group) elected to have their data erased. (DOC 72 kb) [file 12888_2019_2105_MOESM1_ESM.doc]

***Consort flow diagram***

***Follow-Up***

***Enrollment***

Assessed for eligibility (n= 377)

Excluded (n= 56)

  Not meeting inclusion criteria (n= 14)

  Declined to participate (n= 0)

  Other reasons (n= 42)

Analysed ITT (n= 154)
♦ Excluded from all analysis (n= 5)

Lost to follow-up (n= 1)

Discontinued intervention (n= 10)

Allocated to active placebo (n= 159)

♦ Received allocated intervention (n= **148**)

♦ Did not receive allocated (n= 0)

Lost to follow-up (n= 0)

Discontinued intervention (n= 10)

Allocated to ABM (n= 162)

♦ Received allocated intervention (n= **153**)

♦ Did not receive allocated intervention (n= 0)

Analysed ITT (n= 157)
♦ Excluded from all analysis (n= 5)

Randomized (n= 321)

***Allocation***

***Analysis***

Supplemental Figure 1. Flow diagram for enrolment, allocation to active placebo or ABM, follow-up after two weeks, and analyses in accordance with CONSORT (28). Not meeting inclusion criteria= no MDE according to M.I.N.I. Other reasons (n=42)= current or former mania and/or hypomania according to M.I.N.I. Ten participants (5 in each group) elected to have their data erased.
