# Supplementary material for: Effects of Attentional Bias Modification on residual symptoms in depression: a randomized controlled trial
Source: BMC Psychiatry. 2019 May 8;19:141. doi: 10.1186/s12888-019-2105-8 (PMC6505271; doi:10.1186/s12888-019-2105-8)
Supplement: Supplementary file 2 — Supplemental information. (DOCX 22 kb) [file 12888_2019_2105_MOESM2_ESM.docx]

Supplemental information

***Data reduction, compliance rates, and timing of the AB assessment task***

Bias scores were calculated based on median reaction times. We choose to clean data for reaction times below 200- and 2000 ms in accordance with recent literature that have aimed to grasp temporal stability in AB (27). The mean total trials included after applying the filter was 97.25% (,04%) in the placebo group and 97.23% (.02%) in the ABM group and did not differ between the groups [F (1,301) = .003, *p* = .958]. A second filter was used to exclude incorrect responses. The filter affected the placebo group by leaving 99.45% (.007%) and also 99.45% (.008%) in ABM group for further analyse and did not differ between ABM and placebo [F (1,301) = .002, *p* = .962]. A total of 13 participants lacked either pre- or post ABM data that form the basis for the calculation of bias scores. The series mean was used as imputation method for missing data.

Optimal ABM compliance was ensured using a calendar system describing the ABM training that was scheduled individually before the intervention. Compliance rates for the primary outcome measures per protocol (BDI-II and HRSD) were 100%.

Compliance rates (percentage of max 2688 trials) did not differ between ABM (M = 83.5 (14.8)) and placebo (M = 80.0 (21.2)) [F (1,300) = 2.731, *p* = .09]. A post hoc analysis restricted to participants with compliance above 50% (ABM *n* = 147, placebo *n* = 133) did not affect the association between HRSD change and ABM (M = 85.4 (11.5)) versus placebo (M = 86.4 (10.4)) [F (1,269) = 6.265, η^2^ = .02, *p* = .01].

The AB assessment task at baseline was conducted the day before training for all participants. The mean AB assessment time after training (days) at follow up was 2.4 (3.2) and did not differ between ABM and placebo [F (1,301) = .434, *p* = .51].

***Interaction between ABM and stimulus valence- and duration.***

There was a statistically significant interaction between HRSD change x face pairs (positive versus neutral, negative versus neutral, positive versus negative) x intervention (ABM versus placebo) that showed ABM more modification towards positive versus neutral stimuli as compared to the two other valence categories [F (2,309) = 3.278, *p* = .04]. Stimulus duration (500- or 1000 ms) did not interact with HRSD change and intervention [F (1, 309) = .386, *p* = .53].

***Primary outcome per protocol.***

As in the ITT analyses, a repeated ANOVA showed a main effect of ABM as compared to placebo in HRSD scores when the 10 imputed ITT participants were excluded from the analysis [F (1,300) = 6.697, η^2^ = .02, p = .01].
